# Exploring Ohm’s Law: The Randomness of Determinism

**DOI:** 10.3390/e27101058

**Published:** 2025-10-11

**Authors:** Angel Cuadras, Marina Cuadras-Alba, Gaia Cuadras-Alba

**Affiliations:** 1 Electronics Engineering Department (DEEL), Energy, Power and Integrated Circuits (EPIC), Escola Enginyeria de Barcelona Est (EEBE), Universitat Politècnica de Catalunya—BarcelonaTech (UPC), Av. d’Eduard Maristany, 16 Edifici A Campus Besòs, 08029 Barcelona, Spain; 2 Centre d’Estudis Montseny, c/Pallars 410, 08019 Barcelona, Spain

**Keywords:** Ohm’s law, entropy, randomization, determinism, Joule effect, dissipation, relaxation time

## Abstract

Ohm’s law has become ubiquitous in numerous scientific and technical disciplines. Generally, the subject is introduced to students in secondary school as fundamental technical knowledge. The present study proposes a visual model to facilitate the comprehension of Ohm’s law in electron transport in solids to pre-university and university students. The objective is to facilitate students’ comprehension of the correlation between electron movement in solids, as depicted by a current, and the energy of the system, which is introduced by the electric field and the material’s structure. The approach’s originality lies in its novel strategy for describing electron trajectory randomization. This enables the establishment of a relationship between the material’s structure and its resistivity. Moreover, the description of electron transport and scattering processes is presented regarding different types of entropy. It shows that electrons follow the maximum trajectory entropy and that thermal entropy has a quadratic relationship with configurational entropy. The determinism of Ohm’s law is inferred from statistical entropy.

## 1. Introduction

Georg Simon Ohm published his eponymous physical law that relates electrical current, voltage, and resistance in 1827. To commemorate the coming bicentenary, Connelly recently published a thesis that provides a contemporary explanation of the original research [1]. In the 1891 translated edition [2], we identified three interesting ideas. First, Ohm attempted to derive the law in parallel with Fourier’s conduction law. Second, a note from J.C. Maxwell indicated that this was a mistaken approach. Finally, there is a comment in the preface from T.D. Lockwood stating that “many quote Ohm’s law and talk about Ohm’s law, who know little or nothing of Ohm himself, or of his book”. The first idea can be interpreted within the framework of irreversible thermodynamics, wherein Ohm’s law, in conjunction with the Joule effect, elucidates entropy generation in dissipative processes [3]. Conversely, the pervasive use of Ohm’s law in engineering confirms that it has been completely decoupled from the original thermodynamic approach, as Maxwell observed.

The most widely accepted interpretation of Ohm’s law is that the material opposes the flow of an electrical current when subjected to a voltage. However, how the material exhibits this resistance is unclear. At present, electron transport in solid-state physics books [4,5] is described regarding band theory and the Boltzmann equation (Equation (4)). This differential equation of the electron distribution *g* is based on the conservation of momentum of a free electron gas scattering in a solid. In the case that collisions take place, they are introduced with an additional term (∂g/∂t)_coll_, which is difficult to solve unless simplifications are assumed. A common simplification consists of linearizing the term (∂g/∂t)_coll_ using the relaxation time approximation [4]. In addition, to find an analytical solution to (4), it is necessary to assume local thermal equilibrium and that collisions are completely effective in obliterating any information about the nonequilibrium configuration that the electrons may be carrying [4,5].

Table 1 summarizes different expressions (1–4) used to explain this relationship:

- The original approach describes Ohm’s law as a proportionality through a resistor, which must be determined experimentally (Equation (1)).
- When the geometry of the material is introduced, the resistor can be split into resistance, section, and length. Resistance is an intrinsic parameter of the material (Equation (2)).
- Drude proposed a classical model based on electron collisions that satisfactorily explains the relationship between the current density *j* and the electric field *E* through the conductivity σ as *j* = σ *E* and the electron velocity *v* with the mobility *μ* and the electric field as *v* = μ *E* [4,6]. However, the ratio between the current and the field evolves from a constant *R* to a constant *τ* (Equation (3)), which is related to the mean time between collisions of the electrons in the solid. Therefore, the dissipative mechanism is illustrated using an analogy between the resistance and a friction coefficient.
- At present, electron transport in solid-state physics books [4,5] is described regarding band theory and the Boltzmann equation (Equation (4)). This differential equation of the electron distribution *g* is based on the conservation of momentum of a free electron gas scattering in a solid. In the case that collisions take place, they are introduced with an additional term (∂g/∂t)coll, which is difficult to solve unless simplifications are assumed. A common simplification consists of linearizing the term (∂g/∂t)coll using the relaxation time approximation [4]. In addition, to find an analytical solution to (4), it is necessary to assume local thermal equilibrium and that collisions are completely effective in obliterating any information about the nonequilibrium configuration that the electrons may be carrying [4,5].

As mentioned above, there is a relationship between entropy generation and Ohm’s law. Entropy measures the uncertainty of a system. Entropy was historically introduced by Clausius to study the performance of thermal machines, which led to the formulation of the second law of thermodynamics. More recently, Boltzmann related entropy to the available states of a system within the framework of statistical physics. It has been proven that these two approaches are equivalent [7]. This is reasonable, as both expressions define the probability of the thermal system from different points of view: macroscopic in thermodynamics and microscopic in statistical physics. Planck studied the entropy related to the probability of energy availability in oscillators [8], which led to the birth of quantum physics. Shannon generalized Planck’s entropy by averaging probabilities and applied it to information transmission [9]. It is important to understand here that all entropies measure the same thing: the probability of a system’s property. However, it is also worth pointing out that while the entropy of a thermal system measures energy probability, Shannon entropy measures the probability of information transmission quality. More refined general mathematical expressions were derived later, such as those by Tsallis [10] or Rényi [11], of which, Boltzmann, Planck, and Shannon entropies are limit cases.

Ohm’s law is introduced to students in the secondary school curriculum. According to the literature, the physical comprehension of Ohm’s law, as derived from a solid-state approach, is rather complex. The available model conceptualizes electron collisions as random processes such that after each collision, previous information is obliterated. Furthermore, differential equations, founded upon time evolution, are employed for the analysis and prediction of outcomes. While differential equations are a convenient tool for the description of deterministic systems, their application to random processes is typically intractable, as evidenced by the attempts to solve (4). Finally, the reason behind introducing entropy into this context stems from Feynman’s suggestion that Ohm’s law should adhere to a minimum entropy trajectory. However, this approach remains unexplored [12].

Thus, the aim of this study is to propose a straightforward visual model of electron transport in conductors, with the intention of facilitating comprehension among pre-university and university students. The model introduces a novel randomization strategy for the analysis of electron trajectories under the constraints of momentum and energy conservation. This strategy consists of two different phases: first, the possible trajectories of electrons are calculated and second, physics is applied to every electron moving in each trajectory. This approach inverts the conventional sequence of first applying the laws of physics to obtain the trajectory of the electrons and it resembles the Feynman method of summing path integrals using a classical and statistical approach [12]. Here, the underlying reason is to first carry out a statistical simplification of the random trajectories that can lead to a manageable solution of the problem. For convenience, entropy is utilized to evaluate the randomness associated with collisions. This approach recovers Ohm’s original approach to a dissipative system, and it further incorporates entropy concepts that emerged subsequent to Ohm’s contributions. Obviously, given the well-established nature of Ohm’s law within the domain of physics, characterized by its deterministic and causal nature, it is essential that the model incorporates these features.

The objectives of this manuscript are as follows:

- To develop a simplified model that explains electron conduction in solids through Ohm’s law, the Joule effect, and the associated entropy changes.
- To establish a multiphysics framework aimed at helping both pre-university and university students gain a deeper understanding of the role of random processes in physics laws, particularly in Ohm’s law.

The scope of this study is to facilitate a visual comprehension of electron transport for students at the pre-university and university levels. In Spain, the physics curriculum for students around 13 years of age encompasses an introduction to the structure of matter (protons, electrons, and neutrons), energy (mechanical and electrical), and forces (mechanical and electrical). Within the discipline of mathematics, students receive a brief introduction to fundamental probability concepts, encompassing combinatorics and the Gaussian distribution. Following the introduction of mechanical energy, the concept of Ohm’s law is introduced through simple electric circuits. With respect to university undergraduate students, they are familiar with probability theory, with a particular emphasis on the Gaussian distribution. Their studies have encompassed atomic models and the associated concepts of energy and entropy. Furthermore, they possess a comprehensive understanding of fundamental principles, including the conservation of momentum and energy, as well as familiarity with elastic and inelastic collisions.

## 2. Materials and Methods

**Figure 1:**
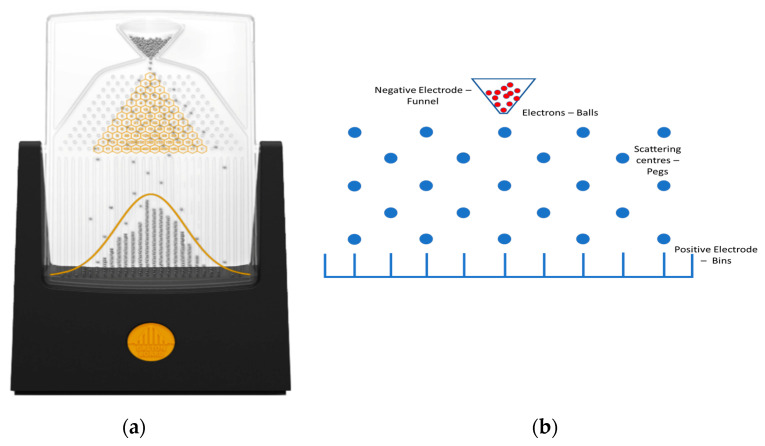
(**a**) Example of a Galton board showing the pegs, falling balls, and Gaussian distribution [13]. (**b**) Analogy of the Galton board with the scattering centre mesh.

The model’s methodology is inspired by the Galton board [13] (Figure 1a). A Galton board consists of a vertical board with interleaved rows of pegs distributed according to a quincunx. Balls are thrown through a funnel at the top and fall down through the peg’s matrix towards a set of bins at the bottom of the board (Figure 1b) [14]. It was proposed to illustrate the central limit theorem in statistics, which states that the distribution of a normalized version of the sample mean converges to a standard normal distribution. When the ball falls and collides with each peg, it randomly falls to the right or left side. Mathematically, this process is described by a binomial distribution. If the number of balls increases, it evolves towards a normal distribution. Several attempts to describe the physical behaviour of the balls’ dynamics can be found in the literature. Hover et al. studied it via conservative classical and quantum Hamiltonian and Gaussian dynamics [15]. They introduced irreversibility into the model but not dissipation. Although it is generally accepted that the dynamics of the balls is stochastic, there have been attempts to model the dynamics using deterministic equations [16,17]. In this case, no experimental proof is provided and simulations do not seem to reproduce the central limit theorem. Computer programs based on deterministic models that lead to Gaussian distributions can the found in repositories.

An extension of the Galton board is the Lorentz gas, which is used for not only for gravity fields, but also for electric fields [18,19]. Chernov et al. used this approach to develop a model for Ohm’s law [20,21], which showed that Gibbs entropy variation in time is related to entropy production and suggested that the linear response was related to the probability distribution of the electrons [22].

**Figure 2:**
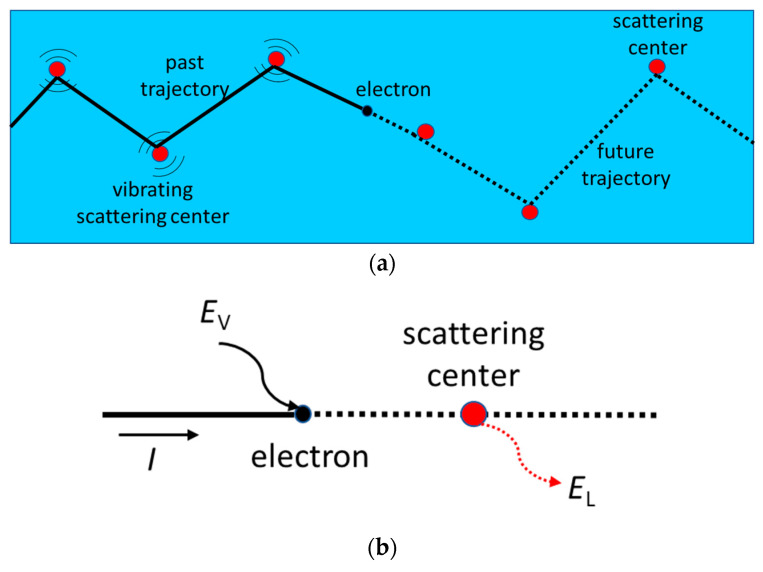
(**a**) Representation of the total electron pre-set trajectory with the electron following it and colliding at the different scattering centres separated an average distance *d*. This figure represents Phase 1 of the method. (**b**) Representation of the energy balance of the electron. It absorbs energy from the electric field *E*_V_ and transfers it to the lattice *E*_L_ when colliding with the scattering centres. The electron travels an average time *τ* between collisions while accelerating in presence of the field.

In our case, it is assumed that the electrons follow a specific trajectory from the starting point (the negative electrode) to the end (the positive electrode). This trajectory depends on collisions within the material, which are randomly distributed along the path. Rather than taking the classical differential approach to study how electrons move from one point to the next within a given time, we adopted an integral approach, considering the final two-dimensional trajectory of each electron (Figure 2a). The critical difference between the two approaches lies in how randomization is performed in the transition from the mechanical to the statistical approach. In mechanical models, such as those explained in Table 1, randomization is carried out for every collision at each instant in time, meaning that previous collision information is obliterated. In our approach, we retain prior information relating to the previous collision for each electron and project it into the future to understand the behaviour of the collective. In the theory of probability, time is less relevant than in classical physics. This means that we do not observe what the electron does at each moment, but rather we take a broader view of what it is expected to do overall. This gives us a general picture of an electron’s behaviour, enabling us to obtain the current trajectory from the sum of all the electrons’ trajectories. Finally, it is worth explaining how the trajectories are set. Electrons are known to move from one electrode to another due to an electric field. The electric field introduces a privileged direction, which breaks the isotropy, favouring collisions in its direction. Unlike pure random collisions such as those in Equation (4), the collisions are random but those aligned in the direction of the field are more probable. Thus, once the trajectory is established, the physics is superposed onto it. The electric field accelerates the electron up to the collision velocity. Figure 2b illustrates how an electron absorbs energy from the field, accelerates in the privileged direction, and collides with the lattice in a random direction, transferring energy to it. In this way, Ohm’s law and the Joule effect are introduced along a preestablished trajectory while preserving momentum and energy conservation.

The following assumptions were considered:

- Each electron and each trajectory are independent of other electrons and trajectories (free electron gas approximation).
- Collisions occur against collision centres, of which, only the impact point, radii, and mass are considered.
- The velocity along the *X*-axis (i.e., in the direction of the electric field) reaches a constant value prior to the next collision, and is referred to as the drift velocity, as it occurs in Ohm’s law and mobility expressions. It is inferred from the acceleration $\dot{v}=v/\tau$; thus, the maximum velocity that can be reach is $v=-eE\tau /m^{*}$ [6]. This simplification implies that no back collisions are considered in this approach but there is no loss of generality.
- The velocity in the *Y*-axis depends on the energy dissipated in each collision and then reabsorbed from the field between collisions.
- The density of scattering centres is a material-specific characteristic, which is characterized by a distance *d*. It is equivalent to the mean free path *λ* = *v*_eq_/*τ* described in Drude’s model [4].
- Collision centres are located at a distance *R* ± *X·d*, where −1 < *X* < 1 is a random number and *d* is chosen to be 20% of *R*. Thus, the collision centres are randomly distributed at a constant average distance *R* ± 20% to account for the fact that scattering centres can move due to thermal vibrations, diffusion, or any other dynamic process.
- Thermal forces are not considered. They are not considered in either Ohm’s law (1) or Drude’s model (3). This approximation is discussed later.
- Two dimensions for collisions are assumed. One dimension would imply either a superconductor or a blocked trajectory by the scattering centre. The extension to three dimensions in these models is straightforward.
- The information track of the electrons is recorded, which is aligned with the privileged direction of the electric field. Electrons do not obliterate past information related to previous collisions.

**Figure 3:**
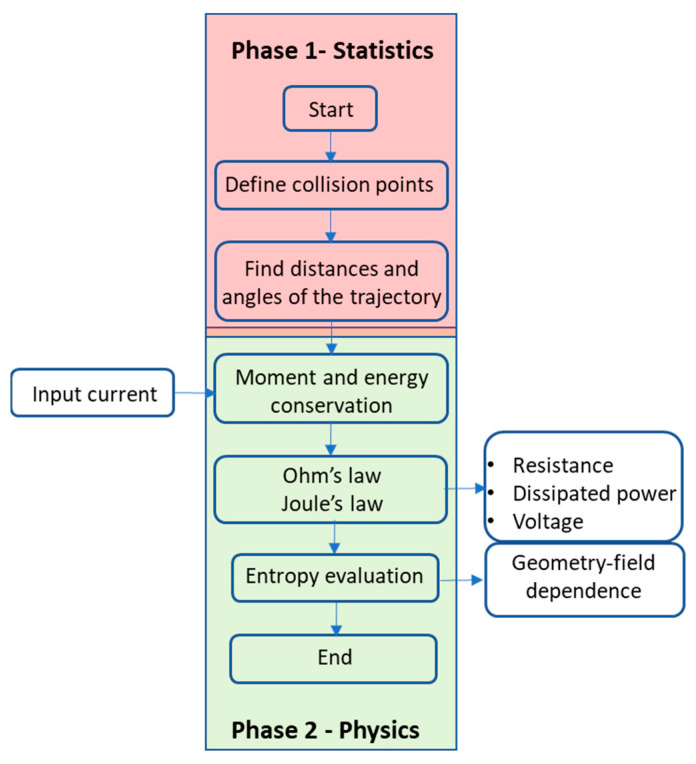
Schematic flow diagram of the simulation processes. In Phase 1, randomized trajectories are created in the direction of the field and across the material. In Phase 2, physics laws are applied to the corresponding trajectories: first, the conservation of momentum and energy, and later, Ohm’s law and the Joule effect. The performance results are obtained from the entropy analysis.

Once the assumptions are fixed, we propose the following steps to understand electronic transport that leads to Ohm’s law, as graphically schematized in Figure 3. Steps 1 and 2 define the statistics of electron trajectories and define Phase 1. Phase 2 defines the physics of the electrons according to conservation laws in step 3 and Ohm’s law and the Joule effect in step 4. Step 5 provides the entropy results of the whole methodology. The numerical simulations are carried out with Matlab R2025A^©^.

Step 1—Location of random centres:

Random centres are located at an increasing *X* and random *Y* using the expression

(5) $$\left(x_{i},y_{i}\right)=\left(x_{i-1},y_{i-1}\right)+\left(d+c\cdot d\cdot (-1{)}^{\alpha }\beta ,d+c\cdot d\cdot (-1{)}^{\gamma }\delta \right)$$

*α* and *γ* are indexes that randomly change between 0 or 1 to have a positive or negative variation with respect to the average distance change *d*. *β* and *δ* are random decimal numbers between 0 and 1. *c* is a constant limiting the variation range around *d*. In our simulations, we choose 0.2 so that the points are located at an average distance *d ± c* in the *X*-axis. With respect to the *Y*-axis, the subsequent collision can only take place within the range ±*c d* from the previous collision. Thus, starting from point (*x*,*y*) = (0,0), we create an array of numbers with an average distance (+*d ±c d*, *±c d*).

Step 2—Simulate the movement of electrons, one by one, by imposing the following:

- A definite number of collisions;
- A definite number of electrons.

Step 3—Analyse the momentum that is transferred to the atoms of the lattice (scattering centres):

(6) $$m^{*}v_{ei}=m^{*}v_{eo}+m_{c}v_{co}$$

Analyse the energy that is transferred to the atoms of the network (collision centres):

(7) $$\frac{1}{2}m^{*}v_{ei}^{2}=\frac{1}{2}m^{*}v_{eo}^{2}+\frac{1}{2}m_{c}v_{co}^{2}$$

where m* is the effective mass of the electron, *m*_c_ is the mass of the collision centre, *v*_ei_ is the input velocity of the electron prior to the collision, and *v*_eo_ and *v*_co_ are the velocities of the electron and the collision centre after the collision. The analysis only considers the energy transfer to the lattice up to the collision instant but does not analyse what occurs in the lattice afterwards.

Step 4—Impose Ohm’s law and the Joule effect on each of the paths that different electrons followed regarding position, momentum, and energy to find the voltage field profile.

Step 5—Evaluate the entropies of the trajectory and energy (details provided in next section).

The current is defined as $I=ne^{-}vA$, where *n* is the electron density per unit volume, *e*^−^ is the charge of the electrons, *v* is the drift velocity of the electrons moving between electrodes, and *A* is the area of the electrodes. It is worth recalling the parallelism between momentum (6) and energy (7) equations and Ohm’s law and Joules’s law:

(8) V=RI

(9) $$E_{diss}=RI^{2}t_{T}$$

where *t*_T_ is the time that the electron takes to complete its trajectory. Current and momentum change linearly with velocity whereas energy is quadratic in both cases. For their part, the electrons lose energy in each collision but after colliding, they take energy back from the electric field to accelerate in the *X* direction until they reach the drift velocity; thus, they always impact the next atom with the same average velocity *v*_x_ (see Figure 2). According to the Joule effect, $E_{diss}=V\cdot I\cdot t_{T}$, where *E*_diss_ is the energy dissipated while traveling along its trajectory that was absorbed from the field, i.e., the original energy that the electron had at the negative electrode. Substituting this into the equation

(10) $$R=\frac{E_{diss}}{n^{2}e^{2}v^{2}A^{2}t_{T}}$$

we obtain the resistance of the material of length *L* and section *A* as a function of dissipated energy and velocity.

### 2.1. Determination of Entropies

The entropy expressions for Shannon entropy, thermal entropy, and statistical entropy used in the results analysis are briefly introduced here.

#### 2.1.1. Shannon Entropy

Shannon entropy is given by

**Figure 4:**
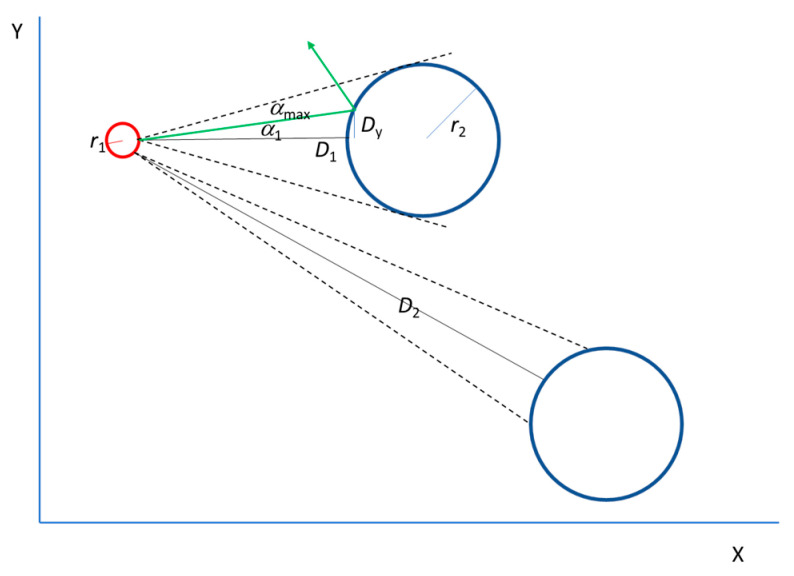
Geometry of the collision between an electron (red circle) and scattering centres (blue circles). The green line represents the trajectory of the electron that collides with particle 1, and *α*_1_ is the angle with respect to the *X*-axis. The black solid lines show the distance between the collision centres and the electron. Angle *α*_max_ corresponds to the maximum angle subtended by the scattering centres.

(11) S=−∑PlnP

where *S* is the entropy and *P* is the probability of an event. In the original work [9], the probability was referred to as information. In our work, however, we simply consider a general probability. In the case of an electron’s trajectory, *P* represents the probability of it taking one path or another at each collision, as in a Galton machine. Different types of probability can be defined (Figure 4). Following the histograms in the results section, the probability of deviating from the *X*-axis *P*_Y_ can be written as

(12) $$P_{Y}=\frac{D_{Y}}{Y_{max}}$$

where *D*_Y_ is the collision distance from the *X*-axis and *Y*_max_ is the total span over the Y-axis. In addition, the geometrical probability *P*_c_ of the subtended angle *α*_c_ (between two consecutive collision points with respect to the *X*-axis) can be considered:

(13) $$P_{c}=\frac{\alpha_{c}}{\pi }$$

*π* is taken as the total angle because no backward collisions are considered in this model. Regarding geometry, it is assumed that the distances between collision points are much larger than their size. In addition, a finite horizon is considered, that is, collisions take place in any direction at a finite distance and no collision-free corridors exist [21] to normalize the probability.

#### 2.1.2. Thermal Entropy

The thermal entropy generated at a resistor due to the conversion of electrical to thermal energy, which is given by [3]

(14) $$S_{i}=\frac{IVt_{T}}{T}$$

where *V* is the voltage drop at the resistor, *I* is the current, *T* is the temperature of the resistor, and *t*_T_ is the integration time.

#### 2.1.3. Statistical Entropy

When a large amount of data is described by Gaussian distribution, the entropy of the distribution represents the degree of dispersion of the samples. Its entropy is given by [23]

(15) $$S_{S}=\frac{1}{2}\ln\left(2\pi \sigma^{2}\right)+\frac{1}{2}$$

where *σ*^2^ is the variance of the Gaussian distribution.

## 3. Results

**Figure 5:**
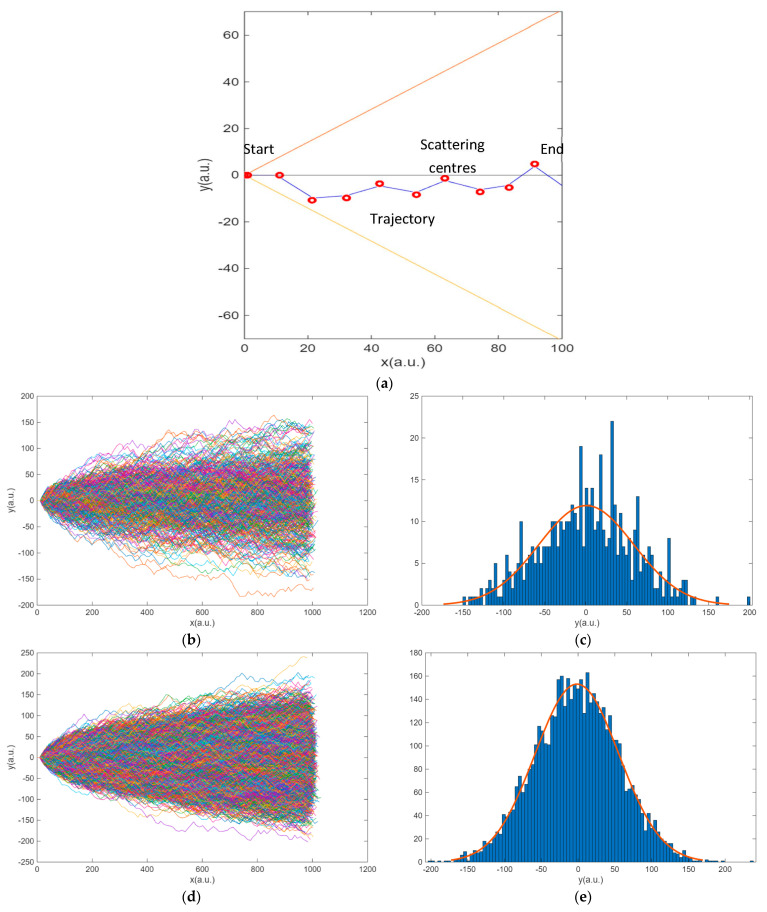
Results of Phase 1. (**a**) Pre-defined trajectory of one electron with random scattering centres from left to right. The orange and yellow straight lines are the maximum *Y* in each position. (**b**) Trajectories of 500 electrons colliding 100 times. (**c**) Histogram of the final positions in the Y-axis in (**b**) with *m* = −1.57 and *σ* = 57.06. (**d**) Trajectories of 5000 electrons colliding 100 times. (**e**) Histogram of the final position in the Y-axis in (**d**) with *m* = −1.57 and *σ* = 57.06.

Following the strategy pointed out in the methodology, the results for the electron transport between electrodes inferred from Phase 1 are illustrated in Figure 5. In (a), the trajectory of a single electron is plotted once the scattering centres have been generated. The electron follows a random trajectory travelling from the negative electrode towards the positive electrode, attracted by the voltage drop. Obviously, one electron does not define Ohm’s law. Simulations with 500 and 5000 electrons are illustrated in Figure 5b,d. Each electron follows a random trajectory between electrodes similar to the one described in (a). This trajectory is independent of the other electrons. Their distributions with respect to the origin are illustrated in the histograms (Figure 5c,e). It is reasonable that as the number of electrons increases, the trajectories are distributed in a Gaussian pattern with respect to the *Y* direction. The mean of this distribution is equal to zero, and the variance is dependent on the geometry.

Once the trajectory of every electron is established, we can evaluate the results of applying the physics laws to the trajectories in Phase 2. The power transferred to the lattice can be estimated from the energy losses at each collision with the scattering centres, as described in Equations (6) and (7):

(16) $$\frac{E_{diss}}{\tau }=I^{2}R=\left(A^{2}n^{2}e^{2}v_{o}^{2}\right)\left(\frac{m^{*}}{ne^{2}\tau }\frac{L}{A}\right)$$

where *τ* is the relaxation time. The dissipated energy at each collision depends on the electron velocity at that collision *v*. Simplifying for one electron,

(17) $$E_{diss}=v_{o}^{2}m^{*}$$

For computation simplification, we have assumed that all collisions take place at the same drift velocity *v*. Thus, we can write the refer the velocity change with respect to the drift velocity as:

(18) $$v_{o}^{2}=kv^{2}$$

where *k* is a constant. A similar result relating both times was found by Chernov et al. [22]. Under these conditions, the dissipated energy is proportional to *k*, and is thus related to the geometry defined by the trajectory.

Determining *k* for each case, we can recalculate *R* using (10) and then find *V*=*R·I*.

(19) $$V=\frac{v_{x}}{e}\frac{d}{\tau }m^{*}\;k=k\frac{m^{*}\cdot {\left(v\cos\alpha \right)}^{2}}{e}$$

where *α* is the angle between the trajectory and the *X*-axis, the voltage is aligned with the *X*-axis. This expression relates the voltage with the geometry of the scattering centres. Thus, we found the connection between the geometry of the material and the electron transport, which matches with the electrical observations: Ohm’s law and the Joule effect.

## 4. Discussion

The implications for entropy shown in Figure 5 are of considerable significance and deserve detailed discussion. We consider implications for characterizing the deterministic behaviour of Ohm’s law, the preferred trajectories of the electrons, the energy transfer to the lattice, and the relationship between the geometry of the material and the opposition to current flow.

**Figure 6:**
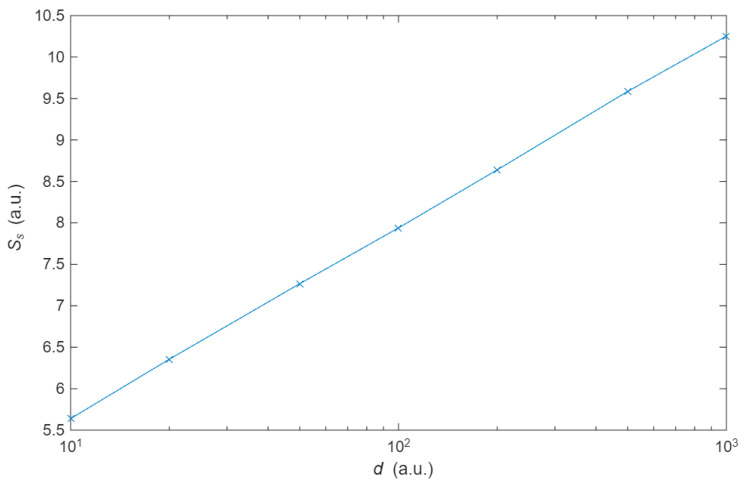
Relationship between distance between collision centres *d* and statistical entropy *S*_s_. *d* is constant for each material. The variance of the distribution is proportional to the distance *σ*= 6.88 *d* + 4.90 (correlation factor *r* = 0.999). When averaged per unit length, the variation becomes constant (a.u. stand for arbitrary units).

First, we consider the profile of the distribution on the histograms. The distribution is found to be Gaussian due to the randomness of the collision. According to Jaynes [24], the Gaussian probability distribution has higher entropy for a given mean and variance, which is useful for describing random processes. The scattering centres were simulated using the Matlab functions *rand* and *randn*, whose function is to generate random numbers that are uniformly and normally distributed, respectively. No discernible difference between the two types of geometries was observed. Furthermore, a Gaussian distribution is also achieved if the scattering centres followed a regular pattern, as in the Galton board shown in the methodology, where the separation between the centres is constant. Thus, the randomness of the collisions is independent of the configuration of the scattering centres. With respect to the properties of the distributions, the mean and variance of the probability distribution are independent of the number of points. It can thus be concluded that the entropy of the probability distribution, as defined for *S*_s_ (13), depends on the distance between scattering centres but not on the number of electrons. This is illustrated in Figure 6, which shows the relationship between the distance between collision centres and the entropy of the probability distribution *S*_s_.

It is also interesting to consider, from the perspective of statistical physics and in the context of Gaussian distributions, the relationship between the mean *<X>* of a magnitude and the fluctuations around the mean *δX* [24]:

(20) $$\frac{\delta X}{<X>}=\frac{\sqrt{n}\sigma }{n\mu }=\frac{1}{\sqrt{n}}$$

From the mean *μ*, the variance *σ* and the number of particles *n* of a system can be obtained. This expression points out that the fluctuations around the mean of the magnitude X decrease as the number of particles increase in a ratio of *n*^−1/2^, that is, systems with a large number of particles can be characterized by the average value <*X*>. Hence, the behaviour of one electron is purely indeterministic, whereas for a system with a large number of electrons, fluctuations decrease, and the system becomes deterministic, an expected behaviour based on Ohm’s law. In the opinion of Chernov et al., the linear response of Ohm’s law deals with probability distributions of the electrons and therefore, a law of large numbers for the macroscopic current should apply [22].

**Figure 7:**
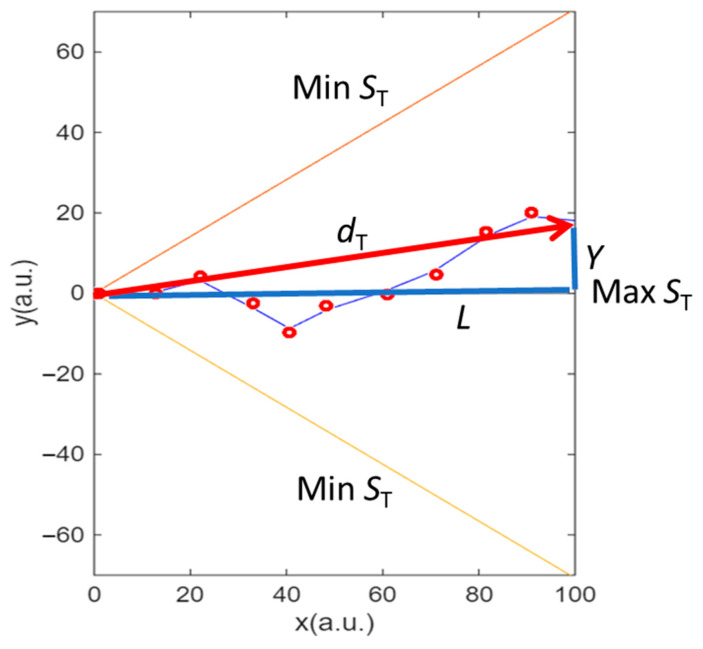
Digitalization of the trajectory by considering the probabilities of the electron moving up and down. The yellow and orange curves define the trajectories with all down or up collisions, respectively. These two curves correspond to the minimum entropy. The blue line over the *X*-axis represents all trajectories with same number of up and down collisions and thus corresponds to the maximum entropy. Regarding the travelled distance, the shorter *d*_T_ path (blue line) corresponds to the trajectory with the maximum entropy. The longest path *d*_T_ (orange and yellow curves) corresponds to the trajectories with the minimum entropy.

Second, as stated in the methodology, the description was inspired by the Galton board. Each trajectory can be described by the angles of collisions. Thus, we can *digitalize* the trajectory by assigning a 1 if the collision deviates the electron upwards and assigning a 0 if the collision deviates the electron downwards. Each trajectory will have the same total number of collisions as imposed by the model. The extreme ones (orange and yellow lines in Figure 7) will be a digital word with all numbers equal (00..00 for the yellow line and 11..11 for the orange line). The trajectories reaching the mean have the same number of up and down collisions and are the most common, as shown in the histograms. We calculate the entropy of the digitalized series as the ratio of the number of times it goes up (*Y* > 0) to the total number of collisions or the number of times it goes up down (*Y* < 0) to the total number of collisions using expression (11) to obtain the trajectory entropy *S*_T_. If all the collisions are in the same direction (orange and yellow lines), the entropy *S*_T_ = 0. If there are the same number of collisions in both directions, *S*_T_ is at the maximum (blue line). This is graphically illustrated in Figure 7.

Thus, in the case of a very large number of electrons, the fluctuations decrease, favouring the most probable trajectories, which are those with the maximum *S*_T_, i.e., the most probable trajectories are those with the greatest trajectory entropy *S*_T_. These two facts allow for the visualization of the movement of electrons: they are constantly colliding to go up and down and move through a kind of channel close to the mean, the one that offers the maximum entropy of the trajectory. The trajectories with the maximum *S*_T_ are parallel to the *X*-axis and they describe the shortest distance between electrodes (Figure 7). The trajectories with the minimum *S*_T_ (orange and yellow lines) are those with the longest distance between electrodes. Electrons prefer the maximum entropy and minimum distance trajectories.

Third, we discuss the thermal entropy in the problem, that is, how the energy of the electrons is transferred to the lattice. The electrons flowing in the electric field collide with the scattering centres, which they transfer energy to. This energy increases the thermal entropy of the lattice. After the collision and before the next collision, the electron reabsorbs energy from the field and starts the same process in the next collision. Thus, we can use the classical expression of entropy generation in electric circuits [3]:

(21) $${\dot{S}}_{i}=\frac{RI^{2}}{T}$$

This expression shows that Ohm’s law is closely related to the Joule effect during current flow through a solid conductor.

**Figure 8:**
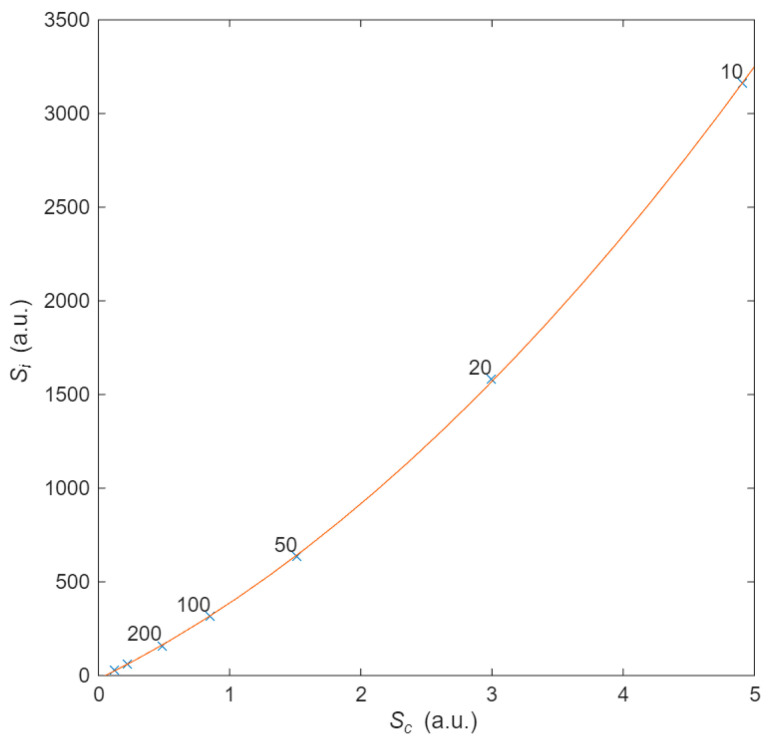
Relationship between thermal entropy and configurational energy. The dots were obtained from simulations with 5000 electrons, 50 collisions, and distances *d* from 10 to 1000. The number labels distinguish the distance between scattering centres. The *S*_i_-*S*_c_ data fit a quadratic curve (in orange) for the different scattering centres distance *d*, proving a causal relationship between the structure of the scattering centres and the energy dissipation.

Finally, following the philosophy of calculating probabilities and entropies, the last analysis relates the configurational entropy of the material with thermal entropy, i.e., we aim to understand how the electron’s energy is dissipated as a function of the material’s structure. To obtain thermal entropy *S*_i_, we integrate (22) over time *t*_T_ for different values of *d*. When *d* increases, in order to maintain a constant *R* for the purpose of comparison, it is necessary to increase *L* in proportion. This dependence of the distance characterizes different materials. The configurational entropy is obtained using (13) from the probability of collision as a function of the angle (calculated using (11)). This entropy provides insight into the interaction between the transport of electrons and the random distribution of the scattering centres. It is also obtained as a function of *d*. Upon eliminating the dependence on *d*, the relationship between thermal entropy and configurational entropy is found. A quadratic relationship was found, as illustrated in Figure 8, for different values of *d*, suggesting causality between them. This quadratic relationship is therefore dependent on the distance between scattering centres through the geometric probability *S*_c_. This final figure illustrates how electrons moving over a geometrical structure, the conductor, in the presence of an electric field obeys the laws of momentum conservation and energy conservation. This relationship clarifies the usual concept “opposition” that a current undergoes when flowing through a resistor. In summary, there is a correlation between entropies, as there is between thermodynamics and statistical physics, and therefore, entropies are causally related [25].

We carried out experimental measurements on resistors to study their degradation mechanisms [26]. Ohm’s law and the Joule effect were satisfied and evolved with degradation, which was explained regarding thermal entropy. However, the best model at the moment was based on unified mechanics theory [27] but it failed to relate the internal structure evolution with the thermal performance. Our present model has solved this issue by relating configurational entropy to thermal entropy.

With respect to the utility of the model for pre-university and university students, it can be useful for the following:

- Introducing entropy linked to probability intuition to understand the real world beyond the limited application to thermal machines. In addition, in contrast to the usual understanding of physics regarding forces, it is possible to describe it regarding energy and entropy, facilitating multiphysics comprehension.
- Introducing collisions (momentum and energy conservation in mechanics). Although the model considers collisions, the analysis can be introduced qualitatively in pre-university courses and quantitatively in university courses.
- Introducing transport properties in solid state courses.

Finally, it is worth mentioning that this probabilistic approach could be useful for a better understanding of probabilistic courses, such as those used in quantum mechanics or artificial intelligence. For our research, it would be helpful in developing battery degradation models. As a basic and novel methodology, it may become suitable for multidisciplinary applications, where different entropies are present, and it is possible to carry out first the entropy simplification and later the physics behaviour.

## 5. Conclusions

The proposed model to describe the transport of nonequilibrium electrons in a resistor under an electric field has provided a simple understanding of Ohm’s law based on the relationship between the entropies present in the problem: the configurational, statistical, and thermal entropies. The following was concluded:

- An electric current in Ohm’s law follows the maximum configurational entropy trajectory, thus becoming a deterministic problem.
- The large number of electrons favour a Gaussian distribution with smaller fluctuations (electrical noise).
- The model satisfies the goal of the study of achieving a simple visual description of Ohm’s law that can be useful to pre-university students. The introduction of probability concepts in elementary physics provides a deeper understanding of physical laws.

## Figures and Tables

**Table 1:** Different models for electron transport in solid conductors. In (1), *I* is the current, *V* is the voltage, and *R* is the resistance. In (2), *ρ* is the resistivity, *L* is the length, and *A* is the area. In (3), m* is the effective mass of the electrons, *n* is the density of the electrons, *e* is the electrical charge of the electrons, and *τ* is the relaxation time. In (4), *g* = *g*(*r*,*k*,*t*) is the distribution that describes the gas of electrons regarding position, momentum, and time; *v* the velocity; *t*, *r*, and *k* are the time, position, and momentum of the electrons; *h* is the Planck constant; *F* is the present electromagnetic force; and the subindex coll is the collision dependence of the electrons with the lattice.

Model			Pros	Cons
Ohm’s law (classical)	I=VR	(1)	Simple to use Experimentally testable	No information about material
Geometry (classical)	R=ρL/A	(2)	A constant is assigned to the material	*ρ* has to be found experimentally
Drude (semiclassical)	$\rho =\frac{m^{*}}{ne^{2}\tau \;}$	(3)	*ρ* is inferred from intrinsic constants of the material	Different definitions of *τ* in the literature Has to be measured experimentally
Solid State (quantum)	$\frac{\partial g}{\partial t}+v\frac{\partial g}{\partial r}+F\frac{1}{\hbar }\frac{\partial g}{\partial k}={\left(\frac{\partial g}{\partial t}\right)}_{coll}$	(4)	Quantum mechanics solution	Based on deterministic differential equations

## Data Availability

Dataset available on request from the authors.

## References

[1] Connelly C.E. (2022). A History of Ohm’s Law Investigating the Flow of Electrical Ideas Through the Instruments of Their Production. Ph.D. Thesis.

[2] Ohm G.S. (1827). The Galvanic Circuit Investigated Mathematically.

[3] Kondepudi D., Prigogine I. (1998). Modern Thermodynamics from Heat Engines to Dissipative Structures.

[4] Ashcroft N.W., Mermin N.D. (1976). Solid State Physics.

[5] Grosso G., Pastori Parravicini G. (2014). Solid State Physics.

[6] Grundmann M. (2006). The Physics of Semiconductors: An Introduction Including Devices and Nanophysics.

[7] Dewar R.C., Lineweaver C.H., Niven R.K., Regenauer-Lieb K. (2014). Beyond the Second Law: Entropy Production and Non-Equilibrium Systems.

[8] Planck M. (1914). Theory of Heat Radiation.

[9] Shannon C.E. (1948). A Mathematical Theory of Communication. Bell Syst. Tech. J..

[10] Tsallis C. (1988). Possible Generalization of Boltzmann-Gibbs Statistics. J. Stat. Phys..

[11] Rényi A. On Measures of Entropy and Information. Proceedings of the Fourth Berkeley Symposium on Mathematical.

[12] Feynman R.P., Leighton R.B., Sands M.L. (2010). The Feynman Lectures on Physics.

[13] Galton Board. https://en.wikipedia.org/wiki/Galton_board.

[14] Galton F. (1889). Natural Inheritance.

[15] Hoover W.G., Moran B., Hoover C.G., Evans W.J. (1988). Irreversibility in the Galton Board via Conservative Classical and Quantum Hamiltonian and Gaussian Dynamics. Phys. Lett. A.

[16] Judd K. (2007). Galton’s Quincunx: Random Walk or Chaos?. Int. J. Bifurc. Chaos.

[17] Mat Daud A.A. (2014). Mathematical Modelling and Symbolic Dynamics Analysis of Three New Galton Board Models. Commun. Nonlinear Sci. Numer. Simul..

[18] Lorentz H.A. (1905). The Motion of Electrons in Metallic Bodies. Proc. R. Acad. Sci. Amst..

[19] Chernov N., Dolgopyat D. (2008). The Galton Board: Limit Theorems and Recurrence. J. Am. Math. Soc..

[20] Chernov N.I., Eyink G.L., Lebowitz J.L., Sinai Y.G. (1993). Derivation of Ohm’s Law in a Deterministic Mechanical Model. Phys. Rev. Lett..

[21] Chernov N., Dolgopyat D. (2007). Diffusive Motion and Recurrence on an Idealized Galton Board. Phys. Rev. Lett..

[22] Chernov N.I., Eyink G.L., Lebowitz J.L., Sinai Y.G. (1993). Steady-State Electrical Conduction in the Periodic Lorentz Gas. Commun. Math. Phys..

[23] Hill T.L. (1986). An Introduction to Statistical Thermodynamics.

[24] Jaynes E.T., Bretthorst G.L. (2003). Probability Theory: The Logic of Science.

[25] Cuadras A., Ovejas V.J., Martínez-García H. (2025). Entropies in Electric Circuits. Entropy.

[26] Cuadras A., Crisóstomo J., Ovejas V.J., Quilez M. (2015). Irreversible Entropy Model for Damage Diagnosis in Resistors. J. Appl. Phys..

[27] Basaran C. (2021). Introduction to Unified Mechanics Theory with Applications.

